# A thoracoscopically resected case of the diverticulum in the middle esophagus

**DOI:** 10.1186/s40792-019-0668-8

**Published:** 2019-07-09

**Authors:** Kentaro Yatabe, Junya Oguma, Soji Ozawa, Kazuo Koyanagi, Akihito Kazuno, Miho Yamamoto, Yamato Ninomiya

**Affiliations:** 1 Department of Gastroenterological Surgery, Tokai University School of Medicine, 143 Shimokasuya, Isehara, Kanagawa 259-1193 Japan; 2 Department of Esophageal Surgery, National Cancer Center Hospital, 5-1-1 Tsukiji, Chuo-ku, Tokyo Japan

**Keywords:** Diverticulum in the middle esophagus, Thoracoscopic surgery, True diverticulum

## Abstract

**Background:**

Approximately 65% of esophageal diverticulum cases are asymptomatic and are found by endoscopic examination. Symptomatic middle esophageal diverticulum requiring surgery is rare. In recent years, endoscopic surgery for middle esophageal diverticulum has been reported, but cases remain few in number, and the surgical indication, surgical procedure, and postoperative results are unknown.

**Case presentation:**

A 41-year-old man had been diagnosed as having a middle esophageal diverticulum based on an upper gastrointestinal contrast examination performed when he was 30 years old. He had not received treatment because he was asymptomatic. Eight months earlier, he experienced chest discomfort after eating and visited our hospital. The diameter of his middle esophageal diverticulum was 47 mm. A gastrointestinal endoscopy revealed a diverticulum in the right wall located 30 cm from the incisor row. The pathological findings of the endoscopic biopsy were atypical epithelium and no malignant findings. We confirmed the function of the lower esophageal sphincter, and the esophageal body peristaltic wave was observed to be normal using high-resolution manometry. We decided to perform a thoracoscopic diverticulectomy based on his symptoms and the possibility of malignancy suggested by the atypical epithelium. Surgery was performed with the patient in a prone position via 4 ports, and intraoperative endoscopy was performed during the surgery. To achieve a complete resection of the diverticulum, threads were placed on the oral and anal sides of the diverticulum, the threads were pulled, and the diverticulum was resected using an automatic suturing device.

A postoperative upper gastrointestinal contrast examination revealed no abnormalities. He was discharged on postoperative day 12.

**Conclusions:**

During thoracoscopic surgery for middle esophageal diverticulum, we think that pulling and separating the diverticulum and confirming the lumen using endoscopy are useful for reducing the risk of postoperative recurrence and stenosis. Few reports of long-term performance after surgery have been made for this procedure. Therefore, we believe that long-term follow-up is necessary.

## Background

Approximately 65% of esophageal diverticulum cases are asymptomatic and are found by endoscopic examination [1]. Symptomatic middle esophageal diverticulum requiring surgery is rare. In recent years, endoscopic surgery for middle esophageal diverticulum has been reported [2, 3], but cases remain few in number, and the surgical adaptation, surgical procedure, and postoperative results are unknown.

## Case presentation

**Fig. 1:**
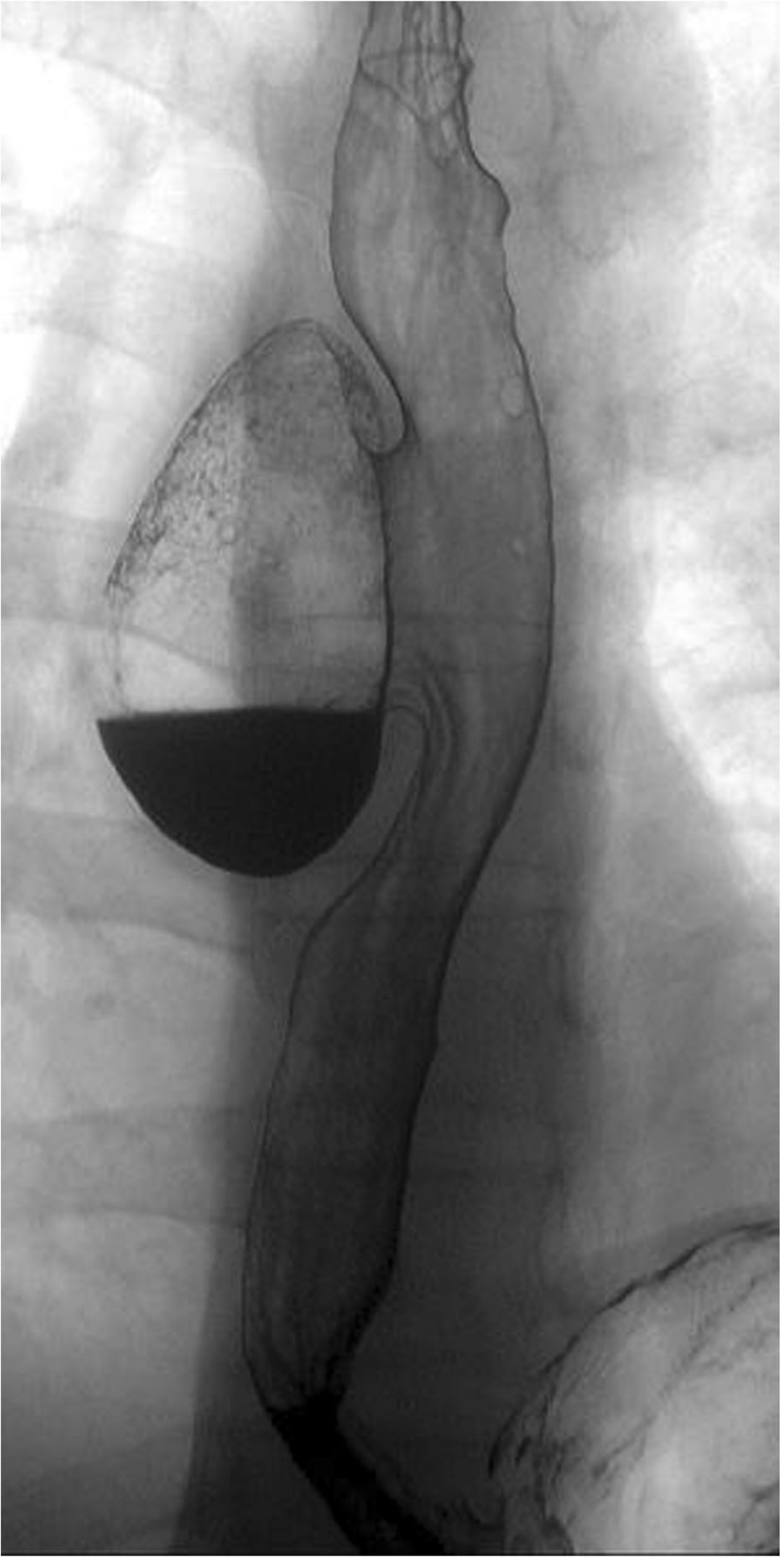
Barium esophagogram of a large diverticulum in the middle esophagus before surgery

**Fig. 2:**
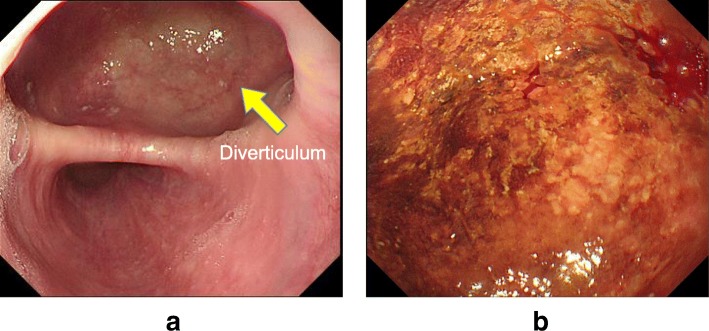
Endoscopic findings before surgery. **a** White light endoscopy revealed a large diverticulum in the middle esophagus. **b** Chromoendoscopy with iodine solution demonstrated multiple small and slightly stained areas at the bottom of the diverticulum

**Fig. 3:**
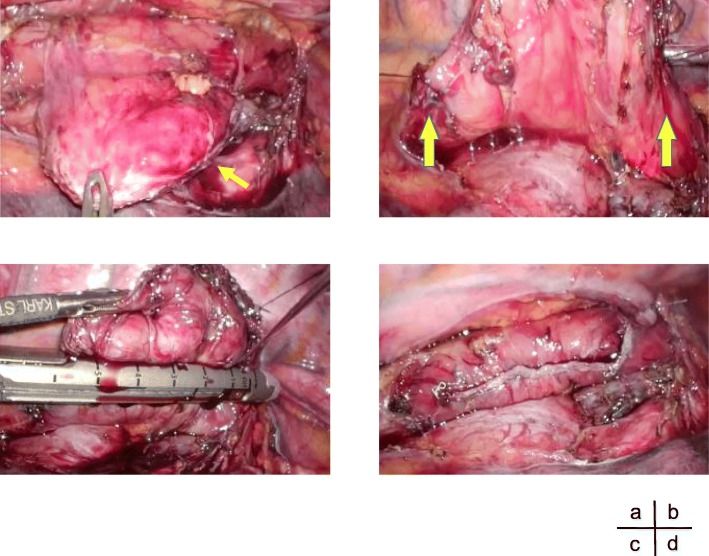
Intraoperative findings. **a** The diverticulum was exposed from the surrounding tissue (arrow). **b** The diverticulum was pulled upwards using anchor threads. **c** A diverticulectomy was performed using a linear stapler. **d** No leakage or bleeding were observed after the diverticulectomy

**Fig. 4:**
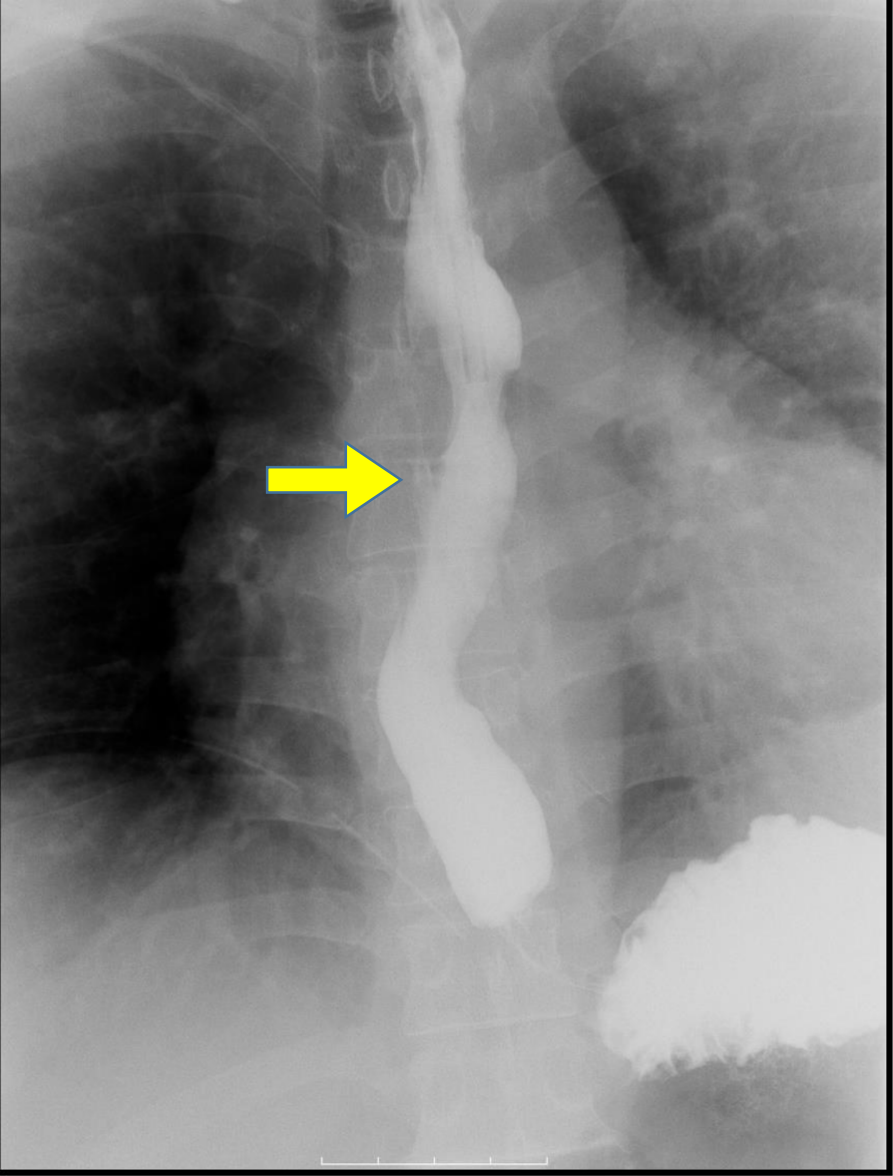
A postoperative upper gastrointestinal contrast examination revealed no abnormalities

**Fig. 5:**
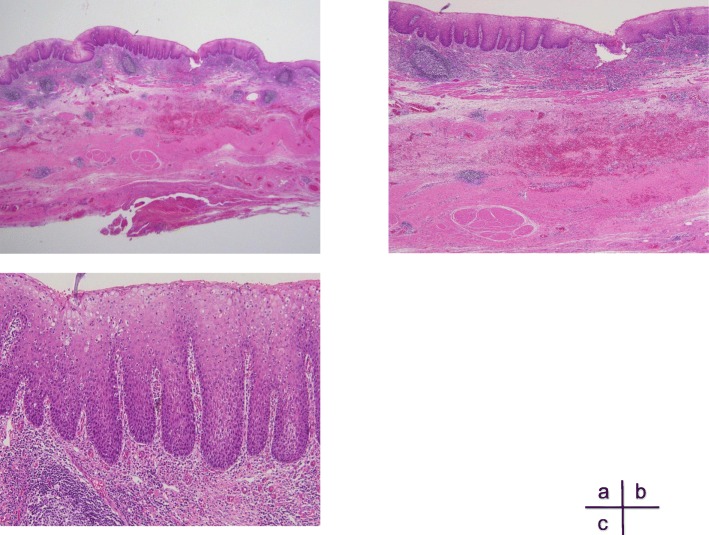
**a**, **b** True diverticulum with a muscular layer and a highly inflamed mucosa. **c** No malignant findings were observed

A 41-year-old man had been diagnosed as having a middle esophageal diverticulum based on an upper gastrointestinal contrast examination performed when he was 30 years old. He had not received treatment because he was asymptomatic. Eight months earlier, he experienced chest discomfort after eating and visited our hospital. His past history is pediatric asthma. The diameter of his middle esophageal diverticulum was 47 mm, and the accumulation of contrast medium was observed in the diverticulum. A gastrointestinal endoscopy revealed a diverticulum in the right wall located 30 cm from the incisor row (Fig. 1), and the diverticulum mucous membrane was partially covered by adherent white matter that was unstained with iodine (Fig. 2), and narrowband imaging which revealed type A. We performed a biopsy, and the pathological findings of the endoscopic biopsy were atypical epithelium and no malignant findings. We confirmed the function of the lower esophageal sphincter, and the esophageal body peristaltic wave was observed to be normal using high-resolution manometry. We decided to perform a thoracoscopic diverticulectomy based on his symptoms and the possibility of malignancy suggested by the atypical epithelium. Surgery was performed with the patient placed in the prone position and four trocars inserted into the right thoracic cavity. A 12-mm trocar was inserted into the fifth intercostal space on the posterior axillary line. Only the left lung was ventilated, and a pneumothorax in the right chest was created using CO2 gas at 6 mm. Twelve-mm trocars were inserted into the seventh and ninth intercostal spaces at the level of the inferior scapular angle. A 5-mm trocar was then inserted into the seventh intercostal space on the posterior axillary line. The thoracoscope was inserted via the 12-mm port in the ninth intercostal space at the level of the inferior scapular angle. The operator used the 12-mm port in the seventh intercostal space on the inferior scapular angle line and the 5 mm port in the seventh intercostal space on the posterior axillary line. The assistant used the 12-mm port in the fifth intercostal space on the posterior axillary line. The endoscope was inserted into the lumen of the upper thoracic esophagus before the patient was placed in the prone position and kept in the esophagus throughout the surgery. Confirmation of the middle esophageal diverticulum was easily achieved, but the area was difficult to exfoliate because the diverticular wall had adhered to the lymph nodes of the trachea bifurcation. To achieve a complete resection of the diverticulum, threads were placed on the oral and anal sides of the diverticulum, the threads were pulled. A thoracoscope was inserted via the 12-mm port in the fifth intercostal space on the posterior axillary line and the surgical stapler was inserted via the 12-mm port placed in the ninth intercostal spaces at the level of the inferior scapular angle, and the diverticulum was resected using two sets of the Tri-Stapler (Fig. 3). A postoperative upper gastrointestinal contrast examination revealed no abnormalities (Fig. 4). Oral intake was initiated on postoperative day 7, and he was discharged on postoperative day 12. The histopathological findings were a true diverticulum with a muscular layer and a highly inflamed mucosa. No malignant findings were seen (Fig. 5). Postoperatively, the patient visited the hospital at 1 month, 3 months, 6 months, and 1 year after the surgery. He underwent gastrointestinal endoscopy and upper gastrointestinal contrast examination 1 year after the surgery. We found no evidence of recurrence of the esophageal diverticulum. We are planning to repeat the upper gastrointestinal endoscopy at 2 years after the surgery.

## Discussion

Traction is generally considered to be the cause of many diverticula, and the esophagus is thought to be pulled outward because of inflammation around the esophagus [4]. In the presently reported case, the histopathological findings revealed a muscular layer in the diverticulum. Although the cause has not yet been elucidated, the intraoperative findings suggested that a lymph node at the tracheal bifurcation might have become enlarged because of inflammation and might have pulled outward.

We performed a thoracoscopic diverticulectomy because the patient was symptomatic and because of the malignant potential of the atypical epithelium at the esophageal diverticulum. We judged that an endoscopic procedure for this lesion might be difficult, mainly because of the technical difficulty of the procedure and the risk of perforation after treatment. Standardized surgical indications for middle esophageal diverticulum have not yet been established. Many reports have suggested that surgery should be performed if any symptoms are present [5, 6]. In recent years, the usefulness of thoracoscopic surgery has been reported as a treatment for middle esophageal diverticulum.

No RCTs of open versus thoracoscopic surgery for middle esophageal diverticulum have been reported. Macke et al. compared the outcomes of 205 patients who received an open thoracotomy and 183 patients who received thoracoscopic surgery and reported morbidity rates of 15% and 23%, respectively, and leakage rates of 7% and 9%, respectively, thereby showing the safety of thoracoscopic surgery [7]. In a thoracoscopic diverticulectomy, the stapler must be inserted and fixed at an appropriate position. However, this technique can minimize bleeding and postoperative pain, and a quick recovery after surgery is expected. Thus, a thoracoscopic diverticulectomy is considered to be a useful treatment for middle esophageal diverticulum resection.

When a diverticulectomy is performed, it is important to reduce the risk of leakage, postoperative recurrence, and stenosis. Based on experience performing Zenker’s esophageal diverticulectomy, the complete resection of the base of the diverticulum was considered to be important to prevent recurrence after surgery. In this case, to separate the diverticula in a secure manner, threads were placed on the oral and anal sides of the diverticulum, and the threads were pulled and to prevent postoperative stenosis, we used an endoscope for the diverticulum resection to check the lumen of each diverticulum. The usefulness of endoscopes during diverticulum removal has been reported [8], and we think that pulling a support thread at the time of diverticulum resection and confirmation of the lumen using intraoperative endoscopy were useful in the present case.

The frequency of carcinogenesis from the esophageal diverticulum has been reported to be 0.3 to 3% [9], and chronic stimulation of the esophageal diverticulum mucosa is considered to be the cause of carcinogenesis. Actually, cancer in the diverticulum is rare, and the mechanism of onset is unknown. For the treatment and observation of the esophageal diverticulum, the possibility of cancer in the diverticulum must be considered. In the present case, the endoscopic examination showed severe inflammation in the diverticular mucosa, but no malignant findings were obtained pathologically. Endoscopic resection has been reportedly performed for superficial cancer of the mucosa of the diverticulum [10, 11]. When performing an endoscopic examination, it is important to thoroughly wash the interior of the diverticulum and to observe it using a special light, magnification, and iodine staining.

## Conclusion

During thoracoscopic surgery for middle esophageal diverticulum, we think that pulling and separating the diverticulum and confirming the lumen using endoscopy are useful for reducing the risk of postoperative recurrence and stenosis. Few reports of long-term performance after surgery have been made for this procedure. Therefore, we believe that long-term follow-up is necessary.

## Data Availability

Data sharing is not applicable to this article, as no datasets were generated or analyzed during the current study.
